# Reassessing Radioactive Iodine Use After Thyroidectomy in Low‐Risk Differentiated Thyroid Cancer: A Systematic Review and Meta‐Analysis

**DOI:** 10.1002/edm2.70293

**Published:** 2026-07-28

**Authors:** Maheen Asif, Mudasar Nisar, Aliza Asif, Makzooma Batool, Rabia Javed Iqbal, Mohammed Hammad Jaber Amin

**Affiliations:** ^1^ Services Institute of Medical Sciences Lahore Pakistan; ^2^ King Edward Medical University Lahore Pakistan; ^3^ Alzaiem Alazhari University Khartoum Sudan

**Keywords:** low‐risk DTC, meta‐analysis, radioactive iodine therapy, treatment response

## Abstract

**Background:**

Radioactive iodine (RAI) therapy is often administered post‐total thyroidectomy in patients with low‐risk differentiated thyroid carcinoma, despite guidelines advising against its routine application. Evidence regarding the efficacy of RAI in reducing recurrence and improving survival in low‐risk differentiated thyroid cancer (DTC) remains inconsistent.

**Methods:**

We conducted a systematic review and meta‐analysis of observational studies and randomized clinical trials that compare RAI versus no RAI in low‐risk DTC patients. Databases such as MEDLINE, Scopus, Cochrane Library, and Google Scholar were searched through December 2025. Outcomes included recurrence, recurrence‐free survival, treatment response, biochemical markers, and side effects. We performed statistical analysis using Review Manager (RevMan) 5.4 with a random‐effects model, reporting odds ratios (OR) with 95% confidence intervals, and significance set at *p* < 0.05. Heterogeneity was assessed using *I* ^2^ statistics.

**Results:**

Ten studies (*n* = 5260) were included in the analysis. No statistically significant difference was observed between the two groups for recurrence (OR 0.66, 95% CI: 0.41–1.08; *p* = 0.10), 5‐year recurrence‐free survival (OR 2.47, 95% CI: 0.63–9.62; *p* = 0.19), or treatment response (excellent response: OR 1.00, 95% CI: 0.52–1.92; *p* = 1.00). Secondary outcomes, including serum thyroglobulin > 1 ng/mL (OR 1.20, 95% CI: 0.00–342.55; *p* = 0.95), elevated thyroglobulin antibodies (OR 0.91, 95% CI: 0.38–2.16; *p* = 0.83), xerostomia (OR 5.63, 95% CI: 0.05–667.24; *p* = 0.48), and dysphonia (OR 0.96, 95% CI: 0.53–1.72; *p* = 0.88), were also not significantly different between groups, although estimates were not precise.

**Conclusion:**

The available randomized and observational evidence does not demonstrate a clear, clinically meaningful benefit of routine RAI ablation in patients classified as low‐risk DTC. These findings should be interpreted with caution due to heterogeneity across studies, variability in outcome definitions, and the predominance of observational data.

## Graphical abstract

Routine radioactive iodine (RAI) after thyroidectomy in low‐risk differentiated thyroid cancer shows no clear benefit in reducing recurrence or improving survival. These findings support a selective, risk‐based approach, avoiding unnecessary treatment and potential adverse effects.

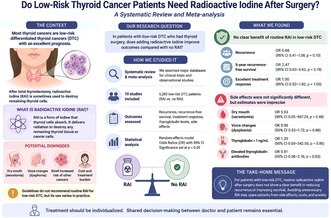

## 1 Introduction

Differentiated thyroid carcinoma (DTC) is the most common endocrine malignancy [1]. Most of these are low‐risk DTC, papillary carcinoma (PTC) being the most common type, accounting for around 84% of cases [2]. The incidence of PTC has been rising, with data from the Surveillance, Epidemiology, and End Results (SEER) program reporting an age‐adjusted incidence rate of 13.5/100,000 population. Clinical decisions are increasingly based on tumour characteristics and individual patient factors [3]. According to the 2015 American Thyroid Association (ATA) guidelines, low‐risk DTC is defined as disease confined to the thyroid without distant metastases, aggressive histology, vascular invasion, or significant lymph node involvement [4]. Accordingly, for patients with low‐risk DTC ranging from 1 to 4 cm, without extrathyroidal extension and without any lymph node metastases, the initial surgical procedure can be either a lobectomy or thyroidectomy. These tumours generally have an excellent prognosis, leading to ongoing debate about the role of adjuvant radioactive iodine (RAI) therapy after thyroidectomy, particularly in improving clinical efficacy. RAI ablation therapy is not usually advised after thyroidectomy for low‐risk DTC patients, particularly those with tumours ≤ 1 cm, confined to the thyroid, and without high‐risk features such as lymph node metastasis or aggressive histology [4].

The thyroid gland selectively takes up RAI, enabling its use for targeted diagnosis and treatment of thyroid disorders, especially after thyroidectomy. However, RAI therapy has also been associated with potential carcinogenicity due to its ionizing radiation effects. A few studies demonstrated that doses above 100 mCi were associated with a dose‐dependent rise in the risk of secondary cancers, highlighting a potential carcinogenic effect of RAI when used excessively [5, 6]. Other potential side effects include xerostomia, dysphagia, sialadenitis, and susceptibility to caries [7].

Previous studies showed that omitting RAI after thyroidectomy in low‐risk DTC patients did not compromise outcomes, with similar recurrence rate [8, 9] and survival rate [10] in the RAI and no‐RAI groups. However, other findings underscore the efficacy of RAI therapy in decreasing recurrence rates and improving long‐term disease control in low‐risk PTC patients [11]. Despite this, the routine use of RAI in low‐risk DTC remains controversial, with some studies showing a reduction in recurrence and improved response while others suggest no significant benefit. The decision to administer RAI in these patients continues to vary among clinicians. Given the inconsistency in outcomes and the potential for overtreatment or undertreatment, a meta‐analysis is needed to clarify the role of RAI in the management of low‐risk DTC. Additionally, recent randomized trials have provided new insights that warrant integration with existing literature. Therefore, an updated and comprehensive meta‐analysis is needed to clarify the clinical benefit of RAI in this population. The aim of this meta‐analysis is to assess the impact of postoperative RAI therapy compared to no RAI on recurrence and clinical outcomes in patients with low‐risk DTC following thyroidectomy.

## 2 Methods

### 2.1 Search Strategy

This meta‐analysis was prospectively registered with PROSPERO (CRD420251087737), conducted according to the Cochrane Handbook for Systematic Reviews of Interventions, and reported following the Preferred Reporting Items for Systematic Reviews and Meta‐Analyses (PRISMA) [12], as well as the Meta‐analysis of Observational Studies in Epidemiology (MOOSE) reporting guidelines [13]. As the study involved analysis of previously published randomized controlled trials (RCTs) and observational studies and did not include direct human subject involvement, ethical approval was not required.

### 2.2 Data Sources and Searching

Two independent reviewers conducted a systematic evaluation of the literature to identify pertinent research published in peer‐reviewed journals. The following databases were searched from inception until 18th December 2025: MEDLINE, Google Scholar, Cochrane Library, and Scopus using a search strategy consisting of relevant keywords and Medical Subject Headings (MeSH). Also, a partial grey literature search and backward citation tracking using references of relevant articles were performed to identify articles further. This included manual screening of reference lists of all included studies and relevant systematic reviews, as well as a forward citation search of key included studies using Google Scholar. Clinical trial registries, specifically https://clinicaltrial.gov, were searched to identify completed but unpublished trials. Conference abstracts from the American Thyroid Association (ATA) annual meetings (2015–2025) were also screened where accessible. Grey literature sources were searched using the same key terms as the primary database search. The search strategy is given in Data S1.

### 2.3 Study Eligibility and Selection

Studies were considered eligible if they followed the following criteria: (1) it was an RCT or observational study; (2) the population included low risk, differentiated thyroid cancer subjects (3) intervention involved radioactive iodine ablation plus total thyroidectomy; (4) the control group underwent total thyroidectomy; and (5) at least one outcome of interest was assessed. Low‐risk DTC was defined in accordance with the 2015 American Thyroid Association (ATA) guidelines [4] as: differentiated thyroid cancer without distant metastases, without macroscopic tumour invasion, without aggressive histology, without vascular invasion, without RAI requiring metastatic disease outside the thyroid bed on post‐treatment scan (if performed), with clinical N0 or N1 disease with fewer than five involved lymph nodes with micrometastasis, with intrathyroidal papillary thyroid carcinomas < 4 cm, or minimally invasive follicular thyroid carcinoma. Studies that reported their cohort as ‘low‐risk’ by the authors' own institutional criteria were accepted if their patient characteristics were broadly concordant with ATA 2015 low‐risk features. Due to the variation in risk classification across the included studies, there is heterogeneity in low‐risk definitions which is explicitly acknowledged as a limitation.

Studies were excluded if they (1) were not peer‐reviewed; (2) were conference abstracts, case reports, review articles, commentaries, or letters; (3) involved animal subjects; or (4) were published in languages other than English. While this restriction may introduce language bias, English‐language journals predominate the indexed literature, and the major trials informing current guidelines are published in English. This limitation is acknowledged in the Limitations section.

### 2.4 Data Extraction

Two researchers extracted data from all the studies using a pre‐defined extraction sheet. The extracted data from each study included the author's name, year of publication, country, number of participants, participant characteristics, and outcomes of interest. The number of observed events was extracted. Data collected by both reviewers were compared, and any disagreements were resolved through discussion with a third author. Inter‐reviewer agreement was high (Cohen's kappa = 0.92), indicating excellent consistency.

### 2.5 Quality Assessment

Two reviewers evaluated the possibility for bias in each study using Cochrane's risk of bias tool (RoB2) [14] for randomized controlled trials and the Newcastle‐Ottawa Scale (NOS) [15] for cohort and cross‐sectional studies. The overall quality of RCTs was assessed and categorized into three classifications based on the randomization method, deviations from prescribed interventions, absent outcome data, outcome evaluation, and reported findings. These categories are classified as high risk, some concerns, or low risk of bias. For a study to be categorized as low risk, it must demonstrate a low‐risk level across all its areas.

The NOS scale assigns ratings ranging from 0 to 9 points for cohort and cross‐sectional research. Index values 0–2 indicate low quality, 3–5 signal moderate quality, and 6–9 denote high quality. The NOS evaluation for cohort studies included the following components: (1) The exposed cohort's representativeness; (2) The unexposed cohort's selection; (3) Exposure verification; (4) Verification that the outcome of interest was not present at the start of the study; (5) Comparability; (6) Outcome evaluation; (7) Follow‐up duration; (8) Adequacy of cohort follow‐up. The NOS evaluation for cross‐sectional studies included the following components: (1) Case representativeness; (2) Sample size; (3) Non response rate; (4) Validation of the screening/surveillance instrument; (5) Comparability; (6) Evaluation of the results; (7) Statistical Analysis. During the review process, any discrepancies were discussed with another author for resolution. Beyond formal NOS scoring, we also assessed key domain‐specific confounders relevant to this review narratively for each observational study. These included: adequacy of T‐stage and nodal status reporting, comparability of baseline thyroglobulin levels between RAI and no‐RAI groups, age and sex distribution, histologic subtype, and whether the treatment allocation appeared to be influenced by clinical risk factors.

### 2.6 Outcome Variables

The study was designed to assess the outcomes in low‐risk thyroid cancer patients treated with radioactive iodine ablation after total thyroidectomy. These outcomes were compared with those who did not receive radioactive iodine ablation after total thyroidectomy. Outcomes included the following:

(1) recurrence, (2) 5‐year recurrence‐free survival, (3) response to treatment, (4) serum thyroglobulin > 1 ng/mL, (5) elevated thyroglobulin antibody, (6) side effects including xerostomia and dysphonia.

### 2.7 Statistical Analysis

We conducted the statistical analysis with the Review Manager (RevMan) software version 5.4 [16] using the random effects model. Odds ratio (OR) was used with corresponding 95% confidence intervals (CIs) as the effect measure for dichotomous variables. ORs were selected as the primary effect measure due to the inclusion of case–control and observational studies, as well as the low event rates across outcomes. Risk ratios or hazard ratios were not consistently reported across studies, limiting their use in pooled analysis. For studies with zero events in one arm, a continuity correction (0.5) was applied automatically by the RevMan software using inbuilt Mantel–Haenszel model. Studies with zero events in both arms were not estimable and were excluded from pooled analysis. A *p*‐value of < 0.05 represented statistical significance. The evaluation of heterogeneity within the studies was performed using a 95% confidence interval and *I* ^2^ statistical analysis. Heterogeneity was considered significant if the *I* ^2^ value was > 50%. Subgroup analyses were performed to explore potential sources of heterogeneity, stratified by study design (randomized controlled trials vs. observational studies) and RAI dose where data were available. These analyses were conducted for outcomes with sufficient studies (recurrence rate and 5‐year recurrence).

## 3 Results

### 3.1 Search Results

**FIGURE 1:**
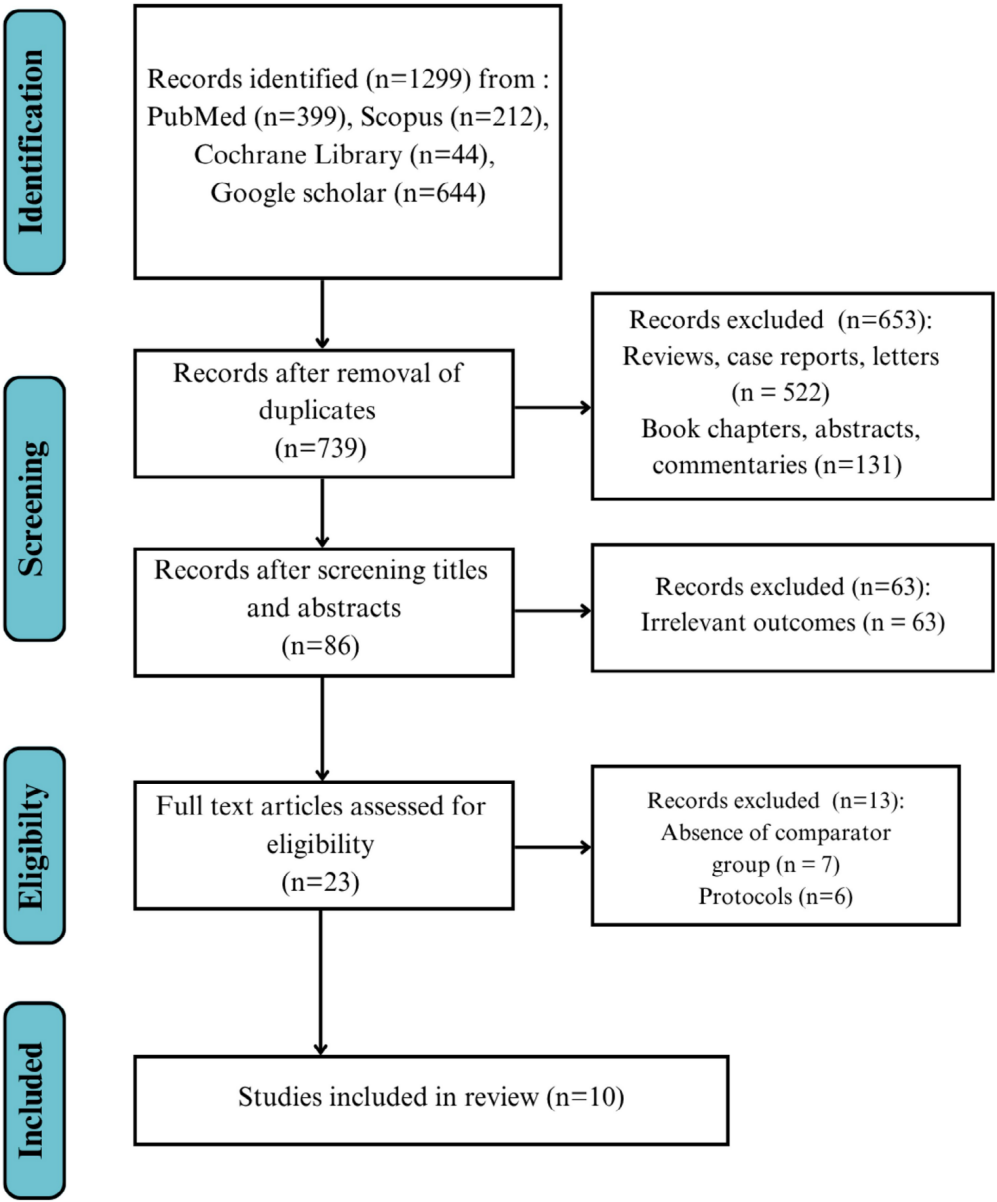
PRISMA flowchart of the study selection process.

The literature search identified a total of 1299 records. Following the removal of duplicates and abstract screening, a total of 23 studies were subjected to full text screening. 7 of them did not have a comparator and 6 were protocols. Ultimately, a total of 2 clinical trials and 8 observational studies with 5260 participants were included in our meta‐analysis [8, 10, 17, 18, 19, 20, 21, 22, 23, 24]. The detailed screening process is shown in Figure 1.

### 3.2 Study Characteristics

Tables 1 and 2 present a summary of the included study characteristics.

**TABLE 1:** Characteristics of included studies.

Study	Study setting	Study design	No of participants	Sex *n* (%)	Age
Durante 2012 [18]	Italy	Retrospective cohort	RAI: 495 No RAI: 290	Male: RAI: 411 (83) No RAI: 251 (86.5)	RAI: 46 (12–77) No RAI: 47 (17–81)
Ibrahimpasic 2012 [20]	MSKCC, USA	Cohort study	RAI: 35 No RAI: 45	RAI: 6 (17) No RAI: 4 (9)	NR
Súss 2017 [23]	Brazil	Retrospective cohort	RAI: 87 No RAI: 102	Female: RAI: 75 (86.2) No RAI: 94 (93.1)	RAI: 43 (19–80) No RAI: 49 (18–86)
Chow 2021 [17]	USA	Survey analysis	RAI: 122 No RAI: 122	Female: RAI: 114 (93.4) No RAI: 113 (92.6)	RAI: 44.6 ± 11.6 No RAI: 43.4 ± 10.4
Leboulleux 2022 [8]	France	Randomized clinical trial	RAI: 389 No RAI: 387	Female: RAI: 319 (82.0) No RAI: 323 (83.5)	RAI: 52.2 ± 13.4 No RAI: 52.6 ± 13.5
Ilera 2023 [21]	Argentina	Prospective cohort	RAI: 58 No RAI: 81	Female: RAI: 48 (82.8) No RAI: 71 (87.7)	RAI: 49 ± 15.14 No RAI: 47.8 ± 15.07
Satapathy 2023 [10]	India	Retrospective cohort	RAI: 1686 No RAI: 388	Females: RAI: 1322 (78.4) No RAI: 297 (76.5)	RAI: 35 (5–84) No RAI: 36 (8–72)
Fatima 2024 [19]	Pakistan	Retrospective cohort	RAI: 92 No RAI: 38	Female RAI: 67 (72.8) No RAI: 31 (81.6)	RAI: 35.61 ± 11.16 No RAI: 32.05 ± 7.91
Xu 2024 [24]	China	Retrospective cohort	RAI: 135 No RAI: 204	Female: RAI: 107 (79.3) No RAI: 163 (79.9)	RAI: 43.0 (32.0–53.0) No RAI: 44.0 (35.0–55.0)
Mallick 2025 [22]	UK	Randomized clinical trial	RAI: 253 No RAI: 251	Female: RAI: 199 (79) No RAI: 191 (76)	RAI: 47 (17–80) No RAI: 48 (17–77)

Abbreviation: RAI = radioactive iodine.

**TABLE 2:** Characteristics of radio iodine ablation and thyroid carcinoma.

Study	Dose of RAI	Follow up duration (Median, range)	Tumour size, mean (cm)	Histology *n* (%)	Positive TgAb *n* (%)	pT stage *n* (%)	Multifocality *n* (%)
Durante 2012 [18]	80 mCi (range 15–104 mCi).	RAI = 6 years (2.5–25) NO RAI = 5 years (2.5–22)	RAI: 12 (0.05–0.4) No RAI: 4 (0.05–2.5)	NR	NR	NR	NR
Ibrahimpasic 2012 [20]^a^	NA	NA	RAI: ≤ 1: 8 (23) 1–2: 13 (37) 2–3: 9 (26) 3–4: 5 (14) No RAI: ≤ 1: 27 (60) 1–2: 10 (22) 2–3: 7 (16) 3–4: 1 (2)	NR	NR	RAI: T1: 21 (60) T2: 14 (40) No RAI: T1: 37 (82) T2: 8 (18)	NR
Súss 2017 [23]	30 mCi	RAI = 49.6 months (4–321) NO RAI = 40.5 months (1–488)	RAI: 1 (0.3–4.0) No RAI: 1 (0.9–9)	Papillary thyroid cancer: RAI: 83 (95.4) No RAI: 94 (93.1)	RAI: 7 (8) No RAI: 7 (6.9)	NR	RAI: 34 (39.1) No RAI: 35 (34.3)
Chow 2021 [17]	NR	Mean years No RAI = 3.1 ± 3.5 RAI = 3.3 ± 3.5	NR	RAI: Papillary: 119 (97.5) Follicular: 2 (1.6) Hurthle cell: 1 (0.82) No RAI: Papillary: 114 (93.4) Follicular: 7 (5.7) Hurthle cell: 1 (0.82)	NR	NR	NR
Leboulleux 2022 [8]	30 mCi (1.1 GBq)	Follow up at 10 months and 3 years	RAI: 1.34 ± 0.36 No RAI: 1.37 ± 0.39	RAI: Papillary: 372 (95.6) Follicular: 13 (3.3) Hürthle cell: 4 (1.0) No RAI: Papillary: 372 (96.1) Follicular: 11 (2.8) Hürthle cell: 4 (1.0)	NR	RAI: pT1aN0: 26 (6.7) pT1aNx: 56 (14.4) pT1bN0: 143 (36.8) pT1bNx: 164 (42.2) No RAI: pT1aN0: 23 (5.9) pT1aNx: 42 (10.9) pT1bN0: 148 (38.2) pT1bNx: 174 (45.0)	RAI: 178 (45.8) No RAI: 156 (40.3)
Ilera 2023 [21]	30 mCi (1.1 GBq)	RAI = 59.5 months (26–91) NO RAI = 61 months (32–82)	RAI: 1.64 ± 0.75 No RAI: 1.4 ± 0.75	RAI: Papillary TC: 53 (91.4) Papillary TC (other): 2 (3.5) Follicular TC: 3 (5.1) No RAI: Papillary TC: 77 (95.1) Papillary TC (other): 1 (1.2) Follicular TC: 3 (3.7)	RAI: 10 (17.2) No RAI: 19 (23.5)	RAI: T1: 46 (79.3) T2: 12 (20.7) No RAI: T1: 72 (88.8) T2: 9 (11.2)	RAI: 15 (25.8) No RAI: 29 (35.8)
Satapathy 2023 [10]^a^	NR	8 years (range: 3–29)	NR	RAI: Papillary TC: 1540 (91.3) Minimally invasive FTC: 146 (8.7) No RAI: Papillary TC: 353 (91.0) Minimally invasive FTC: 35 (9.0)	NR	RAI: T1: 677 (40.2) T2: 714 (42.3) T3a: 295 (17.5) No RAI: T1: 159 (40.9) T2: 169 (43.6) T3a: 60 (15.5)	NR
Fatima 2024 [19]	30–100 mci (*n* = 77) > 100 mci (*n* = 15)	Mean RAI = 2.9 years Mean NO RAI 3.55 years	RAI: 1.96 ± 0.69 No RAI: 1.79 ± 0.70	NR	NR	NR	NR
Xu 2024 [24]	115.5 mCi	RAI = 61.1 months (43.4–73.60) No RAI = 54.1 months (40.5–76.9)	Median (IQR), RAI: 1.4 (0.8–2.0) No RAI: 1.2 (0.6–2.0)	RAI: Papillary: 128 (94.8) Follicular: 7 (5.2) No RAI: Papillary: 192 (94.1) Follicular: 12 (5.9)	NR	RAI: T1: 101 (74.8) T2: 34 (25.2) No RAI: T1: 164 (80.4) T2: 40 (19.6)	RAI: 97 (38) No RAI: 89 (35)
Mallick 2025 [22]^a^	30 mCi (1.1 GBq)	No RAI = 6.8 years (IQR 5.6–8.6) ARI = 6.6 years (4.8–8.5)	NR	RAI: Papillary: 204 (81) Follicular: 38 (15) Oncocytic: 11 (4) No RAI: Papillary: 192 (76) Follicular: 52 (21) Oncocytic: 7 (3)	NR	RAI: T1: 118 (47) T2: 112 (44) T3/T3a: 23 (9) No RAI: T1: 117 (47) T2: 111 (44) T3/T3a: 23 (9)	

^a^ Satapathy 2023 and Mallick 2025 include T3/T3a patients (classified as intermediate‐risk by ATA 2015 guidelines). Ibrahimpasic 2012 includes a substantial T2 proportion. These are acknowledged as potential sources of clinical heterogeneity; NR = not reported, RAI = radioactive iodine.

### 3.3 Risk of Bias Assessment

**FIGURE 2:**
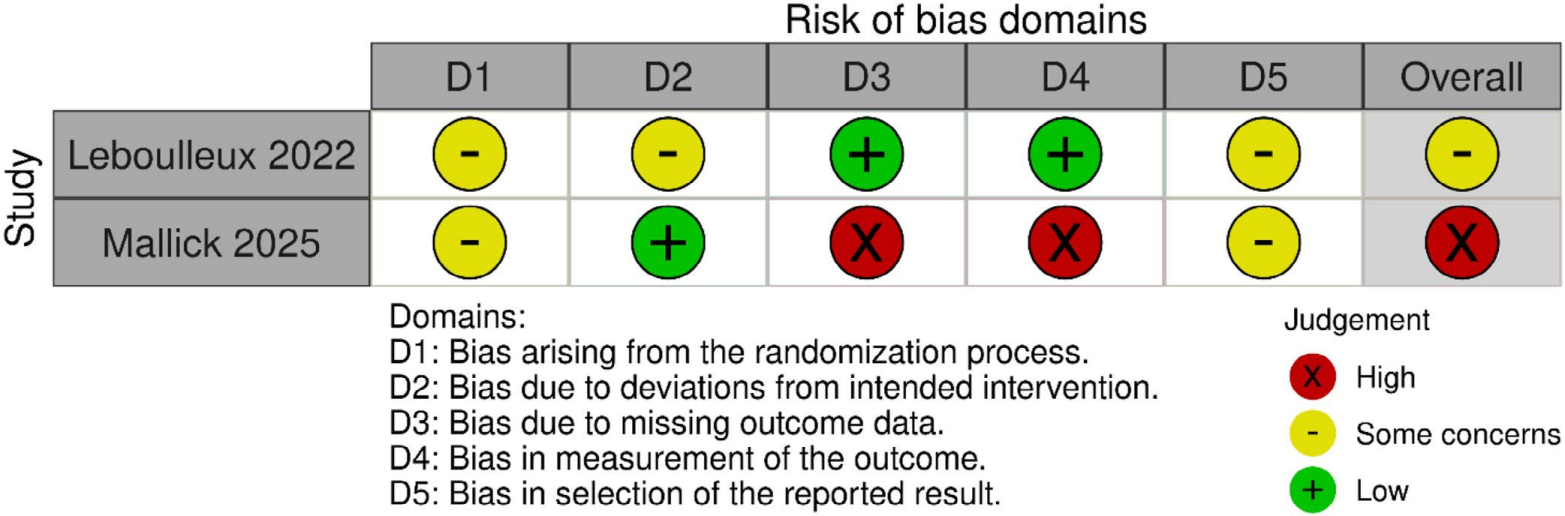
Summary of risk of bias for the included studies assessed using the Cochrane RoB 2 tool (randomized trials). Green cells with ‘+’ indicate low risk of bias; yellow cells with ‘−’ indicate some concerns (RoB 2) or moderate risk; red cells with ‘×’ indicate high risk of bias. Domains assessed include randomization/confounding, deviations from intended interventions, missing outcome data, outcome measurement, and selection of reported results.

The quality evaluation of the included cohort and cross‐sectional studies, utilizing the Newcastle‐Ottawa Scale (NOS), is presented in Tables 3 and 4. The quality of all studies included in this meta‐analysis is high as evidenced by the scale: four studies had 9 stars [10, 18, 21, 23], two studies had 8 stars [19, 24], and two studies had 6 stars [17, 20]. Figure 2 illustrates the risk of bias in the included RCTs as assessed by the ROB2 tool. Among the included studies, one was judged to have some concerns [8] while the other was assessed as having a high risk of bias [22].

**TABLE 3:** Quality assessment for cohort studies.

Sr no.	Study	Selection				Comparability	Outcome			Total
		Representative‐ness of the exposed cohort	Selection of the non‐exposed cohort	Ascertainment of exposure	Outcome not present at start of study		Assessment of outcome	Length of follow‐up	Adequacy of follow‐up	
1.	Fatima et al. [19]	0	*	*	*	**	*	*	*	********
2.	Xu et al. [24]	*	*	*	*	*	*	*	*	********
3.	Súss et al. [23]	*	*	*	*	**	*	*	*	*********
4.	Durante et al. [18]	*	*	*	*	**	*	*	*	*********
5.	Ibrahimpasic et al. [20]	0	*	*	*	*	*	*	0	******
6.	Satapathy et al. [10]	*	*	*	*	**	*	*	*	*********
7.	Ilera et al. [21]	*	*	*	*	**	*	*	*	*********

* Individual asterisks indicate single points earned on the quality assessment scale (e.g., * = 1 point; ****** = 6 points).

**TABLE 4:** Quality assessment of cross‐sectional study.

Sr no.	Study	Selection				Comparability	Outcome		Total
		Representativeness of the cases	Sample size	Non‐response rate	Ascertainment of the screening		Assessment of outcome	Statistical test	
1.	Chow et al. [17]	*	0	0	**	*	*	*	******

* Individual asterisks indicate single points earned on the quality assessment scale (e.g., * = 1 point; ****** = 6 points).

While most observational studies achieved high quality in NOS scores, important domain‐level limitations were identified. None of the included observational studies reported baseline thyroglobulin (Tg) levels as a criteria for treatment allocation, which is clinically significant given that detectable post‐operative Tg is frequently the factor guiding RAI administration. T‐stage distribution was incompletely reported in several studies (Durante 2012, Fatima 2024, Súss 2017), limiting assessment of comparability between groups. Nodal disease status was similarly not reported across most studies. Age and comorbidity data were inconsistently presented, and no study performed multivariable adjustment for major confounders. These gaps indicate that despite high NOS scores, residual confounding by indication remains a meaningful risk across the observational data.

### 3.4 Data Synthesis and Meta‐Analysis

Two clinical trials [8, 22] and eight observational studies [10, 17, 18, 19, 20, 21, 23, 24] investigated the included outcomes. Table 5 provides a concise overview of the meta‐analyses conducted for each outcome.

**TABLE 5:** Outcomes.

Outcomes	Included studies	Participants	OR	95% CI	*p*	Heterogeneity		Model
						*I* ^*2*^	*P*_het_	
Recurrence	7 [10, 18–20, 22–24]	RAI: 2783 No RAI: 1318	0.66	0.41–1.08	0.10	0%	0.50	Random effect
5‐year Recurrence free survival	4 [10, 20, 22, 24]	RAI: 2109 No RAI: 888	2.47	0.63–9.62	0.19	64%	0.04	Random effect
*Response to treatment (RTT)*								
Excellent RTT	3 [8, 21, 23]	RAI: 534 No RAI: 570	1.00	0.52–1.92	1.00	71%	0.03	Random effect
Indeterminate RTT	3 [8, 21, 23]	RAI: 534 No RAI: 570	0.91	0.46–1.80	0.78	64%	0.06	Random effect
Biochemically incomplete RTT	3 [8, 21, 23]	RAI: 534 No RAI: 570	0.54	0.10–2.86	0.47	50%	0.14	Random effect
Structurally incomplete RTT	3 [8, 21, 23]	RAI: 534 No RAI: 570	1.49	0.51–4.36	0.47	0%	0.84	Random effect
Serum Tg > 1 ng/mL	2 [8, 18]	RAI: 884 No RAI: 677	1.20	0.00–342.55	0.95	92%	0.0003	Random effect
Elevated TgAb	2 [8, 22]	RAI: 642 No RAI: 638	0.91	0.38–2.16	0.83	33%	0.22	Random effect
*Side effects*								
Xerostomia	2 [17, 22]	RAI: 375 No RAI: 373	5.63	0.05–667.24	0.48	91%	0.0009	Random effect
Dysphonia	2 [17, 22]	RAI: 375 No RAI: 373	0.96	0.53–1.72	0.88	56%	0.13	Random effect

#### 3.4.1 Recurrence

**FIGURE 3:**
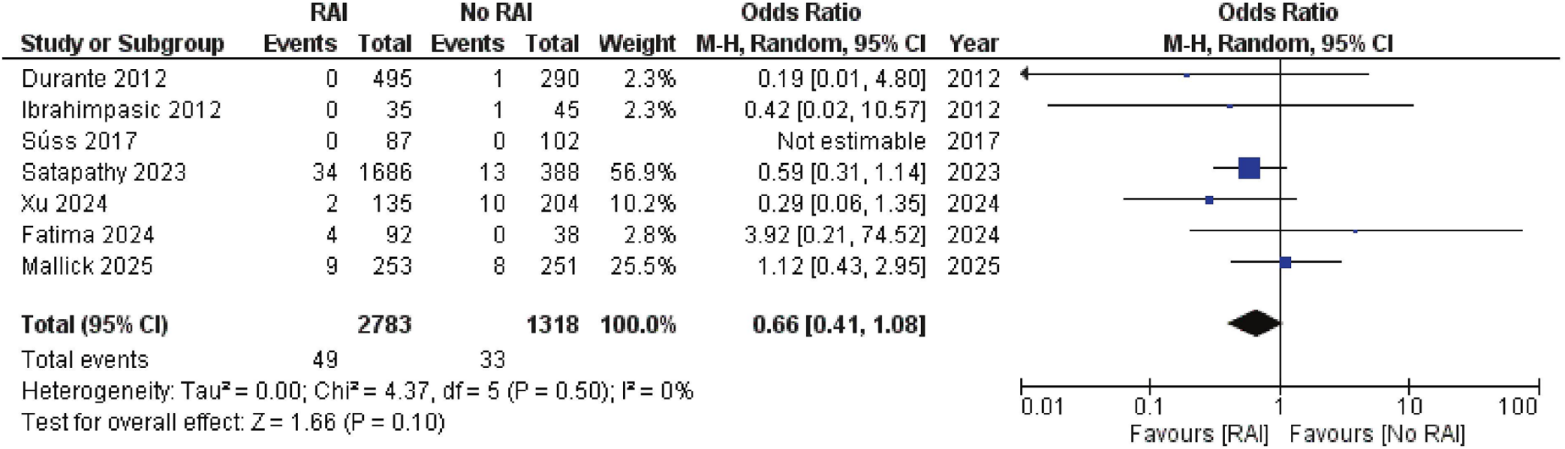
Forest plot of recurrence in RAI and No RAI groups. Odds ratios less than 1.0 favour RAI (reduced recurrence); odds ratios greater than 1.0 favour No RAI. The diamond represents the pooled random‐effects estimate.

This outcome was reported in seven studies [10, 18, 19, 20, 22, 23, 24] with 2783 participants in the RAI group and 1318 participants in the no RAI group. The random effect model was applied. Overall, no statistically significant difference was observed in the recurrence between the two groups (OR: 0.66; 95% CI: 0.41–1.08; *P*: 0.10; Figure 3). No heterogeneity was observed (*I* ^*2*^ = 0%). No difference in effect size or heterogeneity was observed on subgroup analysis based on study design and RAI dose.

#### 3.4.2 5‐Year Recurrence‐Free Survival

**FIGURE 4:**
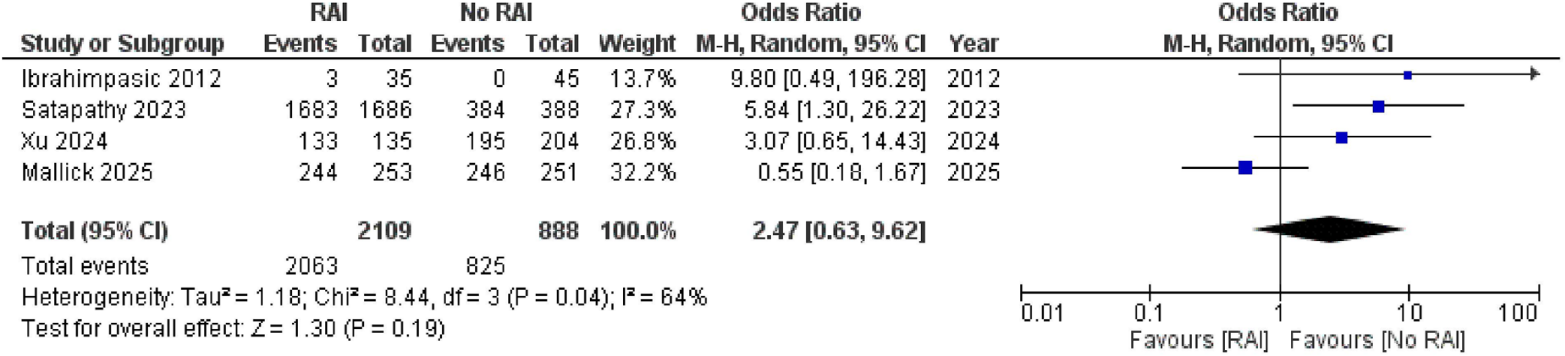
Forest plot of 5‐year recurrence‐free survival in RAI and No RAI groups. Odds ratios less than 1.0 indicate lower odds of maintaining recurrence‐free survival with RAI compared to No RAI; odds ratios greater than 1.0 favour RAI. The diamond represents the pooled random‐effects estimate.

Four of the included studies [10, 20, 22, 24] with 2109 participants in the RAI group and 888 participants in the no RAI group report this outcome. The random effect model was applied. There was no statistically significant difference in the 5‐year recurrence‐free survival between the two groups (OR: 2.47; 95% CI: 0.63–9.62; *P*: 0.19; Figure 4). Heterogeneity was observed to be *I* ^*2*^ = 64%. Subgroup analysis by study design demonstrated that cohort studies showed a significant association favouring RAI (OR 4.70, 95% CI: 1.39–15.97; *I* ^2^ = 0%), whereas the RCT showed no significant effect (OR 0.55, 95% CI: 0.18–1.67).

This stratification resolved heterogeneity within subgroups (*I* ^2^ = 0% for cohort), indicating that study design was a major contributor to the overall heterogeneity (*I* ^2^ = 64.4%).

#### 3.4.3 Response to Treatment (RTT)

**FIGURE 5:**
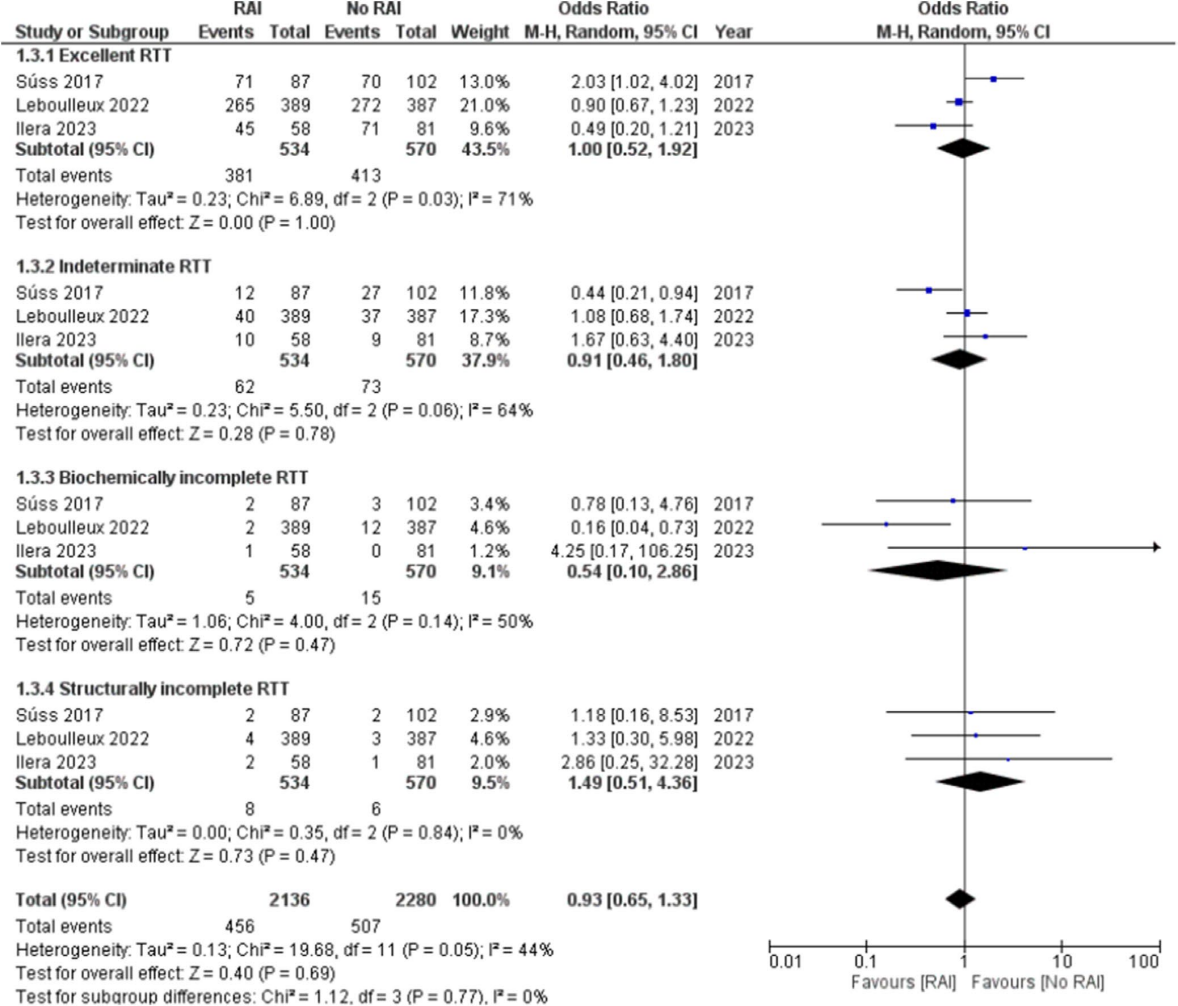
Forest plot of response to treatment in RAI and No RAI groups. Odds ratios less than 1.0 indicate lower odds of achieving the specified treatment response with RAI; odds ratios greater than 1.0 favour RAI. The diamond represents the pooled random‐effects estimate.

*Excellent RTT* was reported in three of the included studies [8, 21, 23] with 534 patients in the RAI group and 570 patients in the no RAI group. The random effect model was applied. No statistically significant difference was observed in the response between the two groups (OR: 1.00; 95% CI: 0.52–1.92; *P*: 1.00; Figure 5). Heterogeneity was observed to be *I* ^*2*^ = 71%. Substantial heterogeneity may be attributed to variations in study design, differences in patient characteristics, and inconsistent definitions of treatment response across studies.

*Indeterminate RTT* was reported in three studies [8, 21, 23] with 534 subjects in the RAI group and 570 subjects in the no RAI group. The random effect model was applied. There was no statistically significant difference between the two groups (OR: 0.91; 95% CI: 0.46–1.80; *P*: 0.78; Figure 5). Heterogeneity was observed to be *I* ^*2*^ = 64%.

*Biochemically incomplete RTT* was reported in three studies [8, 21, 23] with 534 participants in the RAI group and 570 participants in the no RAI group. The random effect model was applied. No statistically significant difference was observed between the two groups (OR: 0.54; 95% CI: 0.10–2.86; *P*: 0.47; Figure 5). No heterogeneity was observed (*I* ^*2*^ = 50%).

*Structurally incomplete RTT* was reported in three studies [8, 21, 23] with 534 subjects in the RAI group and 570 subjects in the no RAI group. The random effect model was applied. There was no statistically significant difference of response between the two groups (OR: 1.49; 95% CI: 0.51–4.36; *P*: 0.47; Figure 5). No heterogeneity was observed (*I* ^*2*^ = 0%).

#### 3.4.4 Serum Thyroglobulin > 1 ng/mL

**FIGURE 6:**
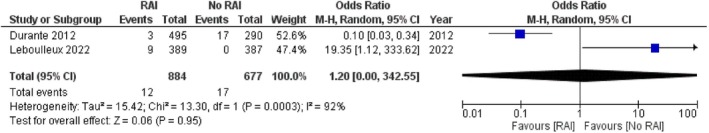
Forest plot of serum Tg > 1 ng/ml in RAI and No RAI groups. Odds ratios greater than 1.0 indicate higher odds of biochemical abnormality in the RAI group. The diamond represents the pooled random‐effects estimate; note the wide confidence interval reflecting sparse data and high between‐study heterogeneity.

Two of the included studies [8, 18] with 884 patients in the RAI group and 677 patients in the no RAI group reported this outcome. The random effect model was applied. The pooled estimate for serum thyroglobulin > 1 ng/mL was OR 1.20 (OR: 1.20; 95% CI: 0.00–342.55; *P*: 0.95; Figure 6), however, the extremely wide confidence interval makes this estimate statistically uninformative, and no meaningful conclusion can be drawn from this outcome. Heterogeneity was observed to be *I* ^*2*^ = 92%. The high heterogeneity likely reflects small sample size, variability in Tg measurement, and differences in follow‐up duration.

#### 3.4.5 Elevated Thyroglobulin Antibodies

**FIGURE 7:**
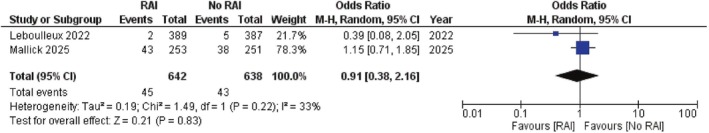
Forest plot of elevated TgAb in RAI and No RAI groups. Odds ratios greater than 1.0 indicate higher odds of biochemical abnormality in the RAI group. The diamond represents the pooled random‐effects estimate; note the wide confidence interval reflecting sparse data and high between‐study heterogeneity.

Two studies [8, 22] with 642 participants in the RAI group and 638 participants in the no RAI group reported this outcome. The random effect model was applied. No statistically significant difference was observed between the two groups (OR: 0.91; 95% CI: 0.38–2.16; *P*: 0.83; Figure 7). Heterogeneity was observed to be *I* ^*2*^ = 33%.

#### 3.4.6 Side Effects

**FIGURE 8:**
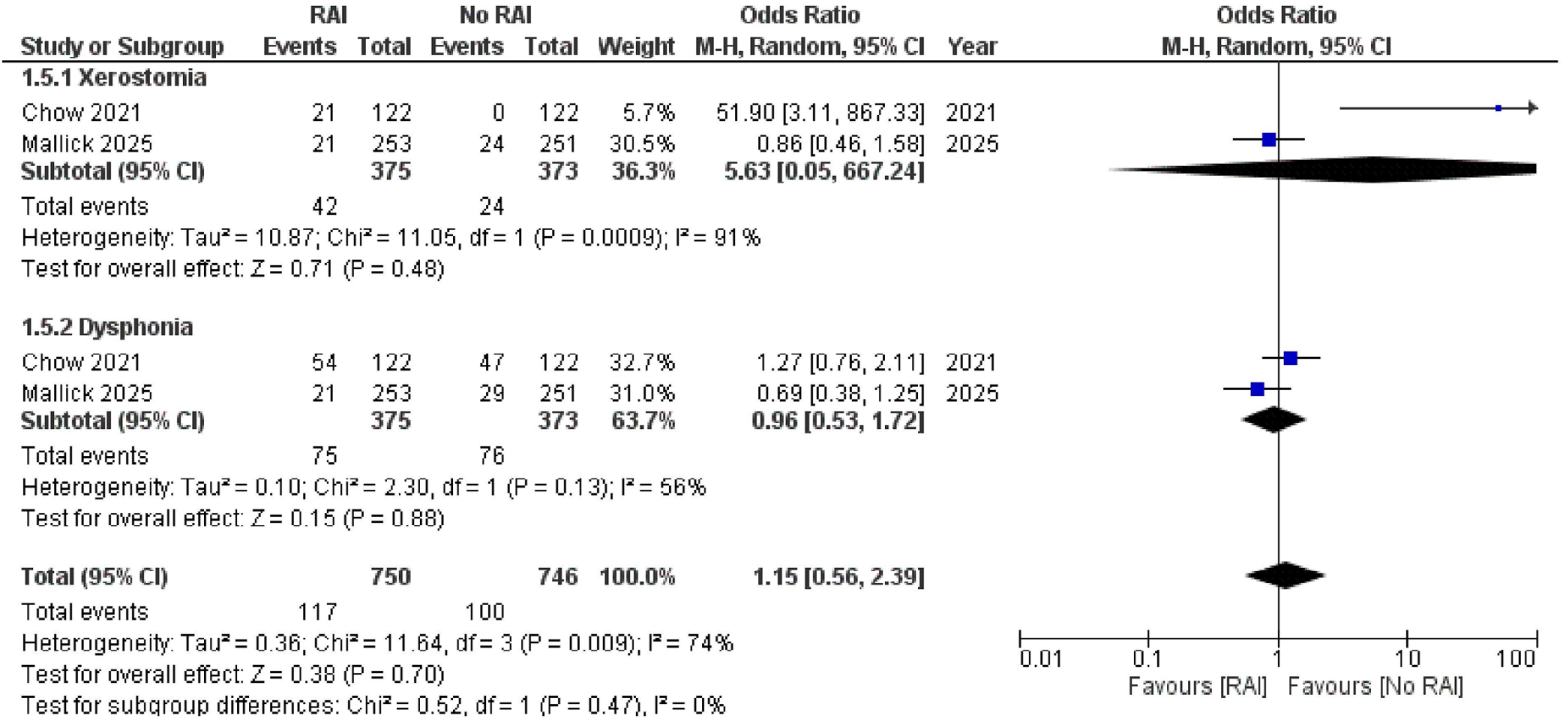
Forest plot of side effects in RAI and No RAI groups. Odds ratios greater than 1.0 indicate higher odds of the respective adverse effect in the RAI group. Wide confidence intervals reflect the small number of contributing studies.

*Xerostomia* was reported in two studies [17, 22] with 375 participants in the RAI group and 373 participants in the no RAI group. The random effect model was applied. The pooled estimate for xerostomia was OR 5.63 (95% CI: 0.05–667.24, *P*: 0.48; Figure 8). Very wide CI makes this result inconclusive and should be interpreted with considerable caution rather than interpreted as evidence of no difference. Heterogeneity was observed to be *I* ^*2*^ = 91%.

*Dysphonia* was reported in two studies [17, 22] with 375 subjects in the RAI group and 373 subjects in the no RAI group. The random effects model was applied. No statistically significant difference was observed between the two groups (OR: 0.96; 95% CI: 0.53–1.72; *P*: 0.88; Figure 8). Heterogeneity was observed to be *I* ^*2*^ = 56%.

## 4 Discussion

Our study demonstrates that the use of RAI ablation following total thyroidectomy in patients with low‐risk differentiated thyroid cancer (DTC) was not significantly associated with a reduction in recurrence and there was no significant improvement in 5‐year recurrence‐free survival. RTT outcomes demonstrated no significant differences between the RAI and non‐RAI groups. Biochemical markers such as serum thyroglobulin levels > 1 ng/mL or elevated thyroglobulin antibodies also showed inconclusive results. Analysis also revealed no statistically significant differences between the two groups for side effects such as dysphonia. The data on serum thyroglobulin levels and xerostomia were too limited and varied to combine reliably. The results had very wide confidence intervals, making them hard to interpret and indicating very low certainty. Pooled estimates and CI of these outcomes do not reflect that the groups are equal; it just means there is not enough evidence to draw a clear conclusion. These findings suggest that routine postoperative RAI may not provide additional benefit for many patients classified as low‐risk DTC, although definitive conclusions remain limited by the available evidence. The role of Tg–guided surveillance is important in interpreting our findings. After total thyroidectomy, serum Tg is the key biomarker for monitoring, and early undetectable or suppressed levels strongly predict long‐term remission in low‐risk differentiated thyroid cancer (DTC). Ibrahimpasic et al. demonstrated that patients with undetectable postoperative Tg derive little additional benefit from radioactive iodine (RAI) [20], while Durante et al. showed that Tg‐based follow‐up without RAI is both feasible and clinically informative [18]. In such cases, surveillance typically involves serial Tg measurements, with or without TSH stimulation. This aligns with the American Thyroid Association (ATA) 2015 framework of dynamic risk stratification [4] which supports personalized Tg‐guided surveillance in low‐risk patients. Despite the imprecision of our Tg estimates, their evidence in existing studies supports the feasibility of omitting RAI in patients with favourable postoperative Tg profiles.

According to 2015 ATA guidelines, the post‐thyroidectomy RAI may be used for several clinical purposes such as remnant RAI ablation, as an additional therapy or for cure of persistent disease. The guidelines gave the risk‐based recommendations for use of RAI and discouraged the use of RAI after thyroidectomy in low‐risk TC patients [4]. Also, guidelines by the National Comprehensive Cancer Network (NCCN) recommended selective rather than routine use of RAI in these patients [25]. This aligns with the findings of our meta‐analysis, which further enhances the idea of unnecessary use of RAI in low‐risk patients after total thyroidectomy. A study by Bilgic et al. observed no difference in recurrence and survival rates between the RAI group and the no RAI group in low‐risk patients undergoing thyroidectomy [11]. Another SEER database study by Weis et al. also concluded similar survival rates following RAI therapy in TC patients, supporting the evidence of insignificant clinical benefit [26]. A study by Schvartz et al. reported a 10‐year overall survival of 95.8% in the non‐RAI group versus 94.6% in the RAI group (*p* = 0.006), and a 10‐year disease‐free survival of 93.1% vs. 88.7% (*p* = 0.001), indicating a modest but statistically significant advantage favouring RAI use [27]. Hence, a strategy reinforcing the de‐escalation of RAI use should be opted for in selective patients, especially when dealing with low‐risk thyroid cancer.

Although our meta‐analysis has shown an insignificant difference in side effects of both groups, RAI is still associated with serious long‐term adverse effects if used for unnecessary time. Reinecke et al. found that the hazard ratio of primary malignancies such as sialadenitis for RAI vs. no RAI ranged from 1.14 to 1.84 across studies while the statistically significant range of relative effect of secondary hematologic malignancies from 1.30 to 2.50 was observed [28]. While cost‐effectiveness and quality of life were not formally analysed in this meta‐analysis, it is worth noting that omitting RAI from thyroidectomy in low‐risk DTC patients also reduces the expenses related to radioisotopes, hospital stays, and patient preparation which highlights its cost‐effectiveness benefit [29].

Based on the results of our analysis, moderate to high heterogeneity was observed in some outcomes that is, 5‐year recurrence‐free survival (*I* ^2^ = 64%) excellent response to treatment (*I* ^2^ = 71%) and serum thyroglobulin > 1 ng/mL (*I* ^2^ = 92%). The variation in demography of patients such as age and gender, and variability in study design and location may have contributed to the heterogeneity in results. Difference in the stage of cancer, tumour size among patients and the definition of low risk also contribute to the causes of heterogeneity in the results.

### 4.1 Strengths and Limitations

Strength of this study lies in comprehensive analysis of the outcomes for both safety and efficacy of RAI in this patient population by sticking to recommended guidelines for conducting meta‐analysis. Persistent evidence of unnecessary use of RAI in low‐risk TC patients further enhances the recommendation by ATA and NCCN guidelines which can also contribute to changes in clinical practice of RAI use. Cost or burden of surgery can be reduced if this unnecessary practice is avoided in selective patients.

Limitations of this study is the inclusion of limited number of studies that is, two RCTS and six observation studies, which increases the risk of bias in the results. Also, high heterogeneity observed in some outcomes limits the interpretation of pooled estimate. The restriction to English‐language publications may have introduced language bias, excluding relevant studies published in other languages such as East Asian, European, or Latin American contexts. Publication bias assessment was not possible because of few numbers of studies included. A formal GRADE certainty‐of‐evidence assessment was not performed because it was not prespecified in the review protocol. The results of this study lack proper assessment of patient related outcomes such as cost‐effectiveness, patient's quality of life and long‐term adverse effects. A notable limitation is the inconsistency in low‐risk definitions across included studies. Notably, Satapathy et al. [10] included T3a cases (17.5% of their RAI cohort), and Mallick et al. [22] included T3/T3a patients (9% of both arms), both of which would be classified as intermediate‐risk by ATA 2015 criteria. Similarly, Ibrahimpasic et al. [20] included a substantial proportion of T2 tumours (40% of the RAI arm), and Súss et al. [23] reported No‐RAI tumour sizes up to 9 cm. These inclusions may dilute the pooled effect of RAI within a truly low‐risk population and contribute to the observed heterogeneity. The inclusion of only two RCTs alongside eight observational studies represents a significant limitation. Although cohort studies suggested improved recurrence‐free survival with RAI, this effect was not observed in randomized trials. This discrepancy is likely attributable to confounding by indication, whereby patients selected to receive RAI differ systematically from untreated patients despite adjustment. In routine clinical practice, physicians may preferentially administer RAI to patients perceived to have higher‐risk pathological features or detectable postoperative thyroglobulin levels, whereas randomized trials minimize these selection biases through random allocation. Consequently, randomized evidence provides a more reliable estimate of treatment effect and suggests that any apparent survival advantage observed in observational studies should be interpreted cautiously.

Sensitivity analyses excluding T2/T3a patients could not be performed because outcome data were not reported separately for these subgroups in the original studies. Individual participant data were unavailable, and excluding entire studies would have unnecessarily discarded large numbers of eligible low‐risk patients. A definitive meta‐analysis of RCT data alone is not feasible at this time given the small number of available trials. The side‐effect analysis was limited to only two studies for both xerostomia and dysphonia, and these findings should be interpreted as preliminary rather than definitive. Large prospective, randomized trials should be designed to investigate these outcomes under suitable protocols. There is a need to investigate patients based on molecular and genetic patterns to help identify low‐risk patients that may benefit from RAI therapy.

## 5 Conclusion

The available randomized and observational data do not demonstrate a clear or clinically meaningful benefit of routine RAI ablation over surgery alone in patients with low‐risk DTC in reducing recurrence, improving 5‐year recurrence‐free survival, or enhancing treatment response. These conclusions should be viewed cautiously because of key limitations such as the predominance of observational data, heterogeneity in outcome definitions, and the availability of only two RCTs. These findings support the guideline recommendations against the routine use of RAI in this population and support a selective, risk‐based approach to postoperative RAI administration. Future research should focus on well‐designed randomized trials with standardized risk stratification, including molecular and genetic profiling. Long‐term follow‐ups should be designed to better evaluate recurrence and treatment‐related toxicity.

## Author Contributions

**Makzooma Batool:** writing – original draft. **Aliza Asif:** writing – original draft, conceptualization, methodology, software, data curation, validation. **Mohammed Hammad Jaber Amin:** writing – original draft. **Mudasar Nisar:** writing – original draft, writing – review and editing, investigation, validation, visualization, methodology, data curation. **Maheen Asif:** writing – original draft, conceptualization, methodology, validation, software, formal analysis, visualization, investigation. **Rabia Javed Iqbal:** writing – review and editing.

## Funding

The authors have nothing to report.

## Ethics Statement

The authors have nothing to report.

## Consent

The authors have nothing to report.

## Conflicts of Interest

The authors declare no conflicts of interest.

## Supporting information


**Data S1:** edm270293‐sup‐0001‐DataS1.docx.

## Data Availability

The data supporting this study's findings are available from the corresponding author upon reasonable request.
