# Supplementary material for: Reassessing Radioactive Iodine Use After Thyroidectomy in Low‐Risk Differentiated Thyroid Cancer: A Systematic Review and Meta‐Analysis
Source: Endocrinol Diabetes Metab. 2026 Jul 28;9(5):e70293. doi: 10.1002/edm2.70293 (PMC13415986; doi:10.1002/edm2.70293)
Supplement: Supplementary file 1 — Data S1: edm270293‐sup‐0001‐DataS1.docx. [file EDM2-9-e70293-s001.docx]

**PubMed**

| **Search number** | **Query** | **Results** |
| --- | --- | --- |
| #1 | "thyroid neoplasms"[MeSH Terms] OR ("thyroid"[All Fields] AND "neoplasms"[All Fields]) OR "thyroid neoplasms"[All Fields] OR ("thyroid"[All Fields] AND "cancer"[All Fields]) OR "thyroid cancer"[All Fields] OR ("thyroid neoplasms"[MeSH Terms] OR ("thyroid"[All Fields] AND "neoplasms"[All Fields]) OR "thyroid neoplasms"[All Fields] OR ("thyroid"[All Fields] AND "carcinoma"[All Fields]) OR "thyroid carcinoma"[All Fields]) | 111450 |
| #2 | "low"[All Fields] AND ("risk"[MeSH Terms] OR "risk"[All Fields]) | 652773 |
| #3 | (("iodine 131"[Supplementary Concept] OR "iodine 131"[All Fields] OR "radioiodine"[All Fields] OR "radioiodines"[All Fields]) AND ("ablate"[All Fields] OR "ablated"[All Fields] OR "ablates"[All Fields] OR "ablating"[All Fields] OR "ablation"[All Fields] OR "ablational"[All Fields] OR "ablations"[All Fields])) OR (("radioactively"[All Fields] OR "radioactivity"[MeSH Terms] OR "radioactivity"[All Fields] OR "radioactive"[All Fields] OR "radioactivities"[All Fields]) AND ("halogenation"[MeSH Terms] OR "halogenation"[All Fields] OR "iodination"[All Fields] OR "iodin"[All Fields] OR "iodinate"[All Fields] OR "iodinated"[All Fields] OR "iodinates"[All Fields] OR "iodinating"[All Fields] OR "iodinations"[All Fields] OR "iodine"[Supplementary Concept] OR "iodine"[All Fields] OR "iodine"[MeSH Terms] OR "iodines"[All Fields])) OR (("iodine 131"[Supplementary Concept] OR "iodine 131"[All Fields] OR "radioiodine"[All Fields] OR "radioiodines"[All Fields]) AND ("remnant"[All Fields] OR "remnant s"[All Fields] OR "remnants"[All Fields]) AND ("ablate"[All Fields] OR "ablated"[All Fields] OR "ablates"[All Fields] OR "ablating"[All Fields] OR "ablation"[All Fields] OR "ablational"[All Fields] OR "ablations"[All Fields])) | 18944 |
| #4 | ("total"[All Fields] OR "totaled"[All Fields] OR "totaling"[All Fields] OR "totalled"[All Fields] OR "totalling"[All Fields] OR "totals"[All Fields]) AND ("thyroidectomy"[MeSH Terms] OR "thyroidectomy"[All Fields] OR "thyroidectomies"[All Fields]) | 14232 |
| #5 | #1 AND #2 AND #3 AND #4 | 399 |

**Google Scholar**

| **Query** | **Results** |
| --- | --- |
| ("thyroid neoplasms"[MeSH Terms] OR ("thyroid"[All Fields] AND "neoplasms"[All Fields]) OR "thyroid neoplasms"[All Fields] OR ("thyroid"[All Fields] AND "cancer"[All Fields]) OR "thyroid cancer"[All Fields] OR ("thyroid neoplasms"[MeSH Terms] OR ("thyroid"[All Fields] AND "neoplasms"[All Fields]) OR "thyroid neoplasms"[All Fields] OR ("thyroid"[All Fields] AND "carcinoma"[All Fields]) OR "thyroid carcinoma"[All Fields])) AND ("low"[All Fields] AND ("risk"[MeSH Terms] OR "risk"[All Fields])) AND ((("iodine 131"[Supplementary Concept] OR "iodine 131"[All Fields] OR "radioiodine"[All Fields] OR "radioiodines"[All Fields]) AND ("ablate"[All Fields] OR "ablated"[All Fields] OR "ablates"[All Fields] OR "ablating"[All Fields] OR "ablation"[All Fields] OR "ablational"[All Fields] OR "ablations"[All Fields])) OR (("radioactively"[All Fields] OR "radioactivity"[MeSH Terms] OR "radioactivity"[All Fields] OR "radioactive"[All Fields] OR "radioactivities"[All Fields]) AND ("halogenation"[MeSH Terms] OR "halogenation"[All Fields] OR "iodination"[All Fields] OR "iodin"[All Fields] OR "iodinate"[All Fields] OR "iodinated"[All Fields] OR "iodinates"[All Fields] OR "iodinating"[All Fields] OR "iodinations"[All Fields] OR "iodine"[Supplementary Concept] OR "iodine"[All Fields] OR "iodine"[MeSH Terms] OR "iodines"[All Fields])) OR (("iodine 131"[Supplementary Concept] OR "iodine 131"[All Fields] OR "radioiodine"[All Fields] OR "radioiodines"[All Fields]) AND ("remnant"[All Fields] OR "remnant s"[All Fields] OR "remnants"[All Fields]) AND ("ablate"[All Fields] OR "ablated"[All Fields] OR "ablates"[All Fields] OR "ablating"[All Fields] OR "ablation"[All Fields] OR "ablational"[All Fields] OR "ablations"[All Fields]))) AND (("total"[All Fields] OR "totaled"[All Fields] OR "totaling"[All Fields] OR "totalled"[All Fields] OR "totalling"[All Fields] OR "totals"[All Fields]) AND ("thyroidectomy"[MeSH Terms] OR "thyroidectomy"[All Fields] OR "thyroidectomies"[All Fields])) | 644 |

**Scopus**

| **Query** | **Results** |
| --- | --- |
| TITLE-ABS-KEY ( (thyroid cancer OR thyroid carcinoma) AND (low risk) AND (radioiodine ablation OR radioactive iodine OR radioiodine remnant ablation) AND (total thyroidectomy) ) | 212 |

**Cochrane**

| **Search**  **number** | **Query** | **Results** |
| --- | --- | --- |
| #1 | (thyroid cancer OR thyroid carcinoma) | 2902 |
| #2 | (low risk) | 82887 |
| #3 | (radioiodine ablation OR radioactive iodine OR radioiodine remnant ablation) | 581 |
| #4 | (total thyroidectomy) | 1211 |
| #5 | #1 AND #2 AND #3 AND #4 | 44 |
